# Preparation and Application of TiO_2_ Nanotube Array Gas Sensor for SF_6_-Insulated Equipment Detection: a Review

**DOI:** 10.1186/s11671-016-1516-4

**Published:** 2016-06-18

**Authors:** Xiaoxing Zhang, Yingang Gui, Xingchen Dong

**Affiliations:** State Key Laboratory of Power Transmission Equipment and System Security and New Technology, Chongqing University, Chongqing, 400044 China; School of Electrical Engineering, Wuhan University, Wuhan, 430072 China

## Abstract

Since Zwilling and co-workers first introduced the electrochemical anodization method to prepare TiO_2_ nanotubes in 1999, it has attracted a lot of researches due to its outstanding gas response and selectivity, making it widely used in gas detection field. This review presents an introduction to the sensor applications of TiO_2_ nanotube arrays (TNTAs) in sulfur hexafluoride (SF_6_)-insulated equipment, which is used to evaluate and diagnose the insulation status of SF_6_-insulated equipment by detecting their typical decomposition products of SF_6_: sulfur dioxide (SO_2_), thionyl fluoride (SOF_2_), and sulfuryl fluoride (SO_2_F_2_). The synthesis and sensing properties of TiO_2_ nanotubes are discussed first. Then, it is followed by discussing the theoretical sensing to the typical SF_6_ decomposition products, SO_2_, SOF_2_, and SO_2_F_2_, which analyzes the sensing mechanism at the molecular level. Finally, the gas response of pure and modified TiO_2_ nanotubes sensor to SO_2_, SOF_2_, and SO_2_F_2_ is provided according to the change of resistance in experimental observation.

## Review

### Introduction

Titanium dioxide (TiO_2_) nanotube has been widely researched due to its distinguished properties, including high surface-to-volume ratios, high surface activity, strong catalytic activity, and high ultraviolet light adsorption and heat conductivity [1–3]. It has been used in fields such as industrial manufacturing, aerospace, ocean exploring, environmental protection, resource development, and medical diagnose [4–7]. To meet the increasing high requirement for gas detection, TiO_2_ nanotube gas sensors are investigated for qualitative or quantitative gas detection. However, the detection response, selectivity, and accuracy of pure TiO_2_ are limited for different gases detection. To improve its detection performance, the most used methods are morphology control and surface modification [2, 8, 9], aiming to increase the effective reaction surface and active site. For common gas detection such as O_2_, H_2_, SO_2_ and H_2_S, the highest detection limit has even reaches parts per million level [10–14].

Sulfur hexafluoride (SF_6_) insulating gas possesses outstanding arc quenching and insulation performance, which is the most used filled gas in gas-insulated equipment, such as gas-insulated switchgear (GIS), gas-insulated lines (GIL), and gas circuit breaker (GCB) [15–17]. However, SF_6_ gas will inevitably decomposes to various typical decomposition components: SO_2_, SOF_2_, SO_2_F_2_, etc. under partial discharge and disruptive discharge (surface flashover and creeping discharge) when insulation defects occurs in production and long term operation process [18–20]. The insulation defects in SF_6_-insulated equipment show great influence on the stability of entire insulated system. On the one hand, the dielectric strength of filled insulated gas obviously reduces under discharge because of the decomposition of SF_6_. On the other hand, the decomposition components (low-fluorine sulfides) corrode the surface of SF_6_-insulated equipment with the action of trace water and oxygen in equipment [21]. Besides, most of the insulation defect-induced discharge is hard to be found by the inspection workers as the discharge is always unsustainable. Therefore, online detection method, which assesses the insulation status automatically in real time, becomes an effective to solve the detection difficulty [22–24]. However, the current detections methods: ultra high frequency (UHF) method, transient earth voltage (TEV) method, ultrasonic method, and fluorescence detection method are easily affected by the environmental interference signal [25–28]. Thanks to the distinguished anti-interference and high detection precision properties of gas sensors detection method, online gas detection becomes a new breakthrough for insulation status assessment of SF_6_-insulted equipment.

In this paper, we will review the achievements in the filed using TiO_2_ nanotubes for three typical SF_6_ decomposition components: SO_2_, SOF_2_, and SO_2_F_2_ detection. Firstly, pure morphology of TiO_2_ nanotubes is prepared by adopting different preparation methods. In addition, the surface modification of TiO_2_ nanotubes is analyzed by experimental study to enhance the gas detection response. Secondly, the gas sensing properties are discussed to analyze the gas detection mechanism by theoretical studies. Finally, the gas sensing property to three typical SF_6_ decomposition products is discussed by theoretical and experimental studies. Meanwhile, the influence factors such as gas concentration and sensing time are presented in detail.

### Synthesis of TiO_2_ Nanotubes

**Fig. 1:**
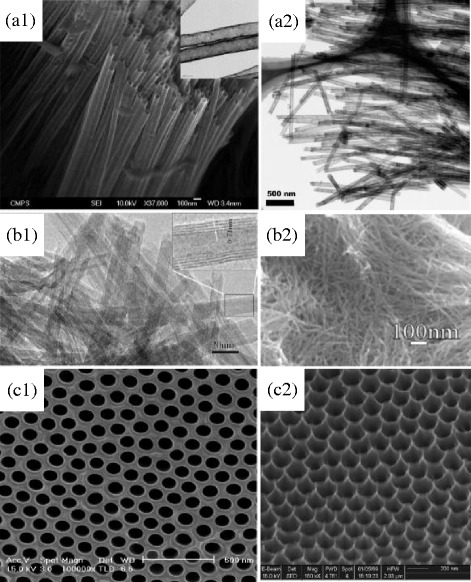
TiO_2_ nanotubes synthesized by different methods: **a1**, **a2** Assisted-template method, **b1**, **b2** hydrothermal treatment method, and **c1**, **c2** anodization method

Assisted-template method is one of the effective methods to synthesize TiO_2_ nanotubes, as the synthesized TiO_2_ nanotubes reported by Hyunjung et al. shown in Fig. 1a [29]. In terms of the preparation process, it is fabricated by filling the nano-structural unit into the hole of different templates, including anodic aluminum oxide (AAO) template, high polymer template, porous silica template and mesoporous zeolite, etc. AAO template is one of the most used method to synthesize of TiO_2_ nanotubes [30]. Firstly, AAO is prepared by anodization method to serve as the template to produce the polymer mold. Then, amorphous TiO_2_ is electrochemically deposited to the hole of the AAO template. After high-temperature anneal, the amorphous TiO_2_ turns to TiO_2_ nanotubes, which shares the same diameter with the hole of AAO template. Finally, the TiO_2_ nanotubes are received by dissolving the AAO template with strong alkali solution. Martine et al. successfully fabricated different kinds of metal nanotubes: TiO_2_, Co_3_O_4_, MnO_2_, WO_3_, and ZnO nanotubes by this method [31]. In other study, Peng et al. fabricated bamboo-shaped TiO_2_ nanotubes with an average diameter of 100 nm by upright dipping manner [32]. The bamboo-shaped nanotubes consist of many hollow compartments that are separated by TiO_2_ layer.

Due to the features of simple and low-price preparation, hydrothermal treatment method is an effective method to synthesize TiO_2_ nanotubes in large scale industrial production. The morphology of the produced TiO_2_ nanotubes are usually composed by small diameter, thin wall, and large surface area nanotubes, and the nanotubes are usually unordered and intertwined as shown in Fig. 1b [33]. For the synthesis of TiO_2_ nanotubes, firstly, mixing the TiO_2_ nanoparticles with strong alkali solution under high temperature and high pressure, the single-layer nanosheets of TiO_2_ appear in the treatment process curl from one dimension to two and three dimensions, which is similar with the formation mechanism of carbon nanotubes. After the chemical reactions, the TiO_2_ nanotubes is received by ion-exchange and anneal. Finally, the powder-like TiO_2_ nanotubes is obtained by centrifuged by centrifugal machine after neutralizing the strong alkali solution with weak acid solution. According to the report of Weng et al. in 2006 [34], TiO_2_ nanotubes, with an external diameter of around 8 nm and a wall thickness of about 1 nm, was synthesized by hydrothermal treatment method.

The anodization method is one of the effective ways to synthesize ordered alignment and aspect radio TiO_2_ nanotubes as shown in Fig. 1c [35]. The anodic Ti foil dissolve (metal corrosion or electropolishing) to Ti metal cation under the action of electrolyte and electric field. On the one hand, the produced Ti metal cation reacts with the O^2−^ (produced by water electrolysis) and forms a TiO_2_ oxidation film on the surface of Ti foil, resulting in the increase of resistance. Therefore, the formation rate of TiO_2_ oxidation film decreases. On the other side, the produced TiO_2_ oxidation film is dissolved by the electrolyte. Under the combined action of formation and dissolution, nanotube arrays synthesize on the surface of Ti foil. The nanotube morphology depends on many influence factors: applied voltage, electrolyte composition, and pH value. In 2000, Grimes et al. presented that an aligned and organized TiO_2_ with an average tube diameter from 25 to 65 nm nanotubes fabricated by anodization method [36]. In addition, Grimes reviewed the fabrication, properties of highly ordered TiO_2_ nanotube arrays made by anodic oxidation of titanium in fluoride-based electrolytes [37]. They found that the length of TiO_2_ tends to be longer in the weak acid electrolytes, and the wall of TiO_2_ nanotubes becomes smoother in organic electrolytes.

Due to the outstanding properties, high surface area, ordered alignment, and morphology adjustability of TiO_2_ nanotubes, it shows great potential in gas detection. However, the pure TiO_2_ nanotubes are hard to meet the high detection requirement because of its limitation in gas response, detection range, response time, and operational temperature. In this regard, great efforts have been made to extend the gas detection properties. Currently, a few modification methods: metal decoration, doping, semiconductor composites are introduced to improve the gas sensing properties [38, 39]. Comparing the different modification methods, metal decoration is one of the most effectively method to significantly enhance the gas response of TiO_2_.

Metal decoration can be achieved in two different ways: metal nanoparticles decoration and metal ions decoration. For metal nanoparticles decoration, the metal nanoparticles are loaded or deposited on the surface of pure TiO_2_ nanotubes to change the electron distribution in TiO_2_ nanotubes system. For metal ions decoration, the ions are intruded into TiO_2_ lattice, resulting in a trace of metal ions take the place of Ti atoms in the TiO_2_ lattice by physical and chemical approaches. The decorated metal atoms improve the gas sensing properties by changing the electron distribution and energy band. Xiaoxing et al. synthesized Pt atom modified TiO_2_ nanotubes in H_2_PtCl_6_·6H_2_O (1 g/L) and H_3_BO_3_ (20 g/L) electrolytes by pulsed electrodeposition [40], which show good response to SF_6_ decomposition products: SO_2_, SOF_2_, and SO_2_F_2_. Shahin el al. reported that the Au- and Ag-decorated TiO_2_ nanotubes sensor exhibited a large resistance variation in the presence of very small quantities of H_2_ gas at 25 °C [31].

### Theoretical Studies

In order to evaluate and diagnose the insulation status of SF_6_-insulated equipment, it is necessary to precisely detect each kind of decomposition products of SF_6_: SO_2_, SOF_2_, and SO_2_F_2_, respectively. However, these three kinds of gas products usually appear at the same time when discharge occurs in SF_6_-insulated equipment, leading to the cross interference between different products. Therefore, a lot of researches have been made to enhance the gas selectivity while improving the gas response.

**Fig. 2:**
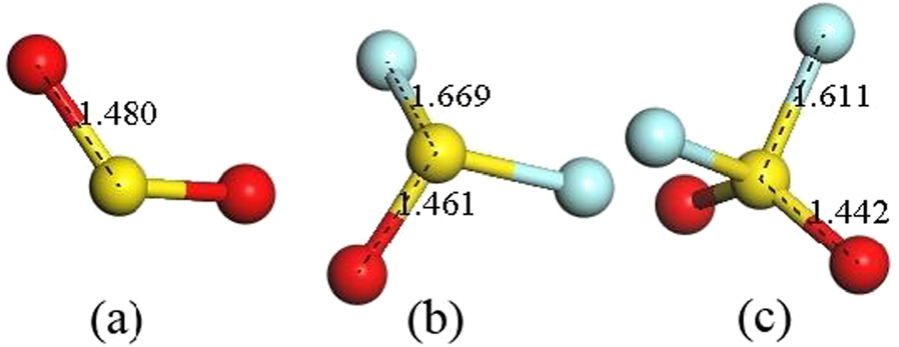
Structure of the gas molecules: **a** SO_2_ molecule, **b** SOF_2_ molecule, and **c** SO_2_F_2_ molecule. S, O, and F atoms are respectively shown in *red*, *yellow*, and *cyan*

**Fig. 3:**
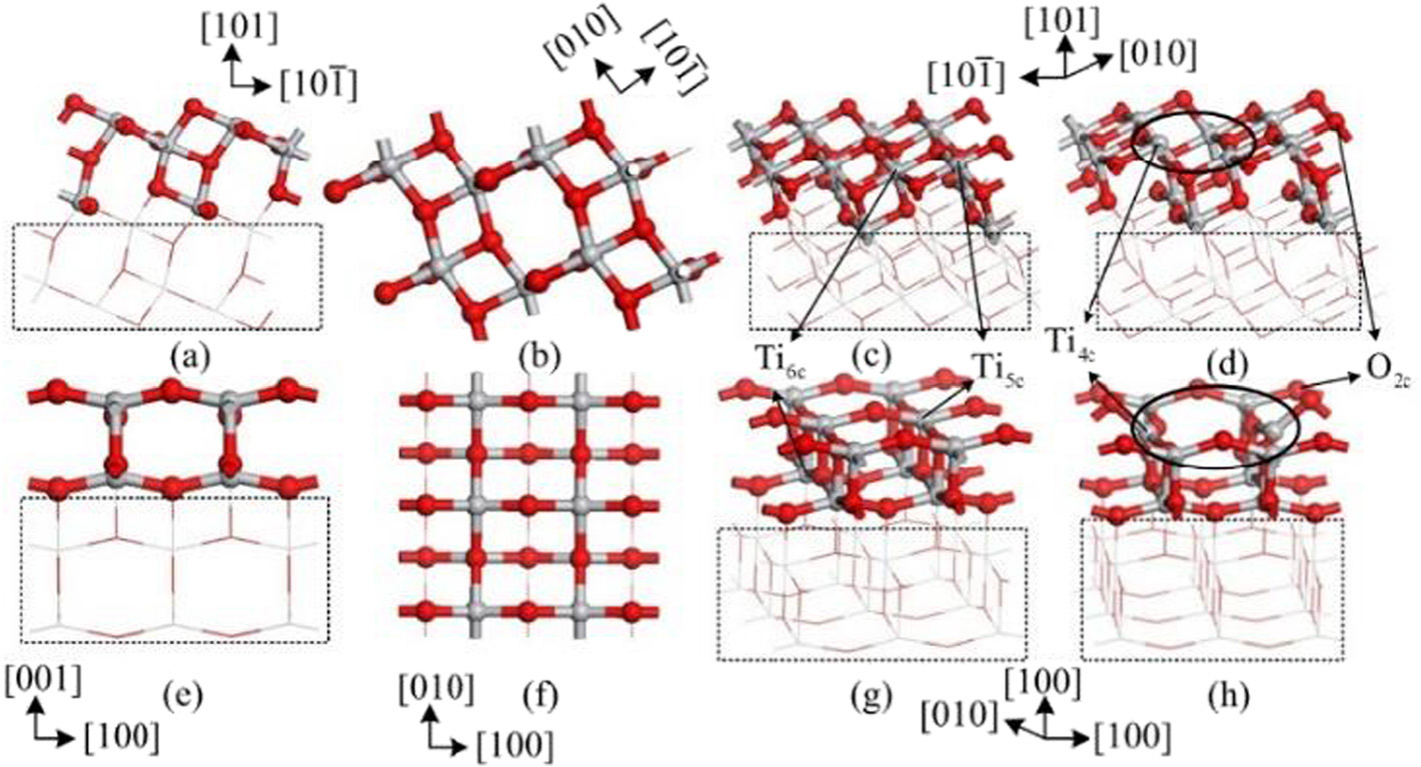
Four kinds of surfaces. **a**–**c** The views of (1 0 1) perfect surface. **d** (1 0 1) defect surface. **e**–**g** The views of (0 0 1) perfect surface. **h** (0 0 1) defect surface. Ti and O atoms are shown in *gray* and *red*, respectively. Ti_6c_, Ti_5c_, and O_2c_ are marked by *arrows*, and oxygen vacancy sites are marked by *ellipse*

**Fig. 4:**
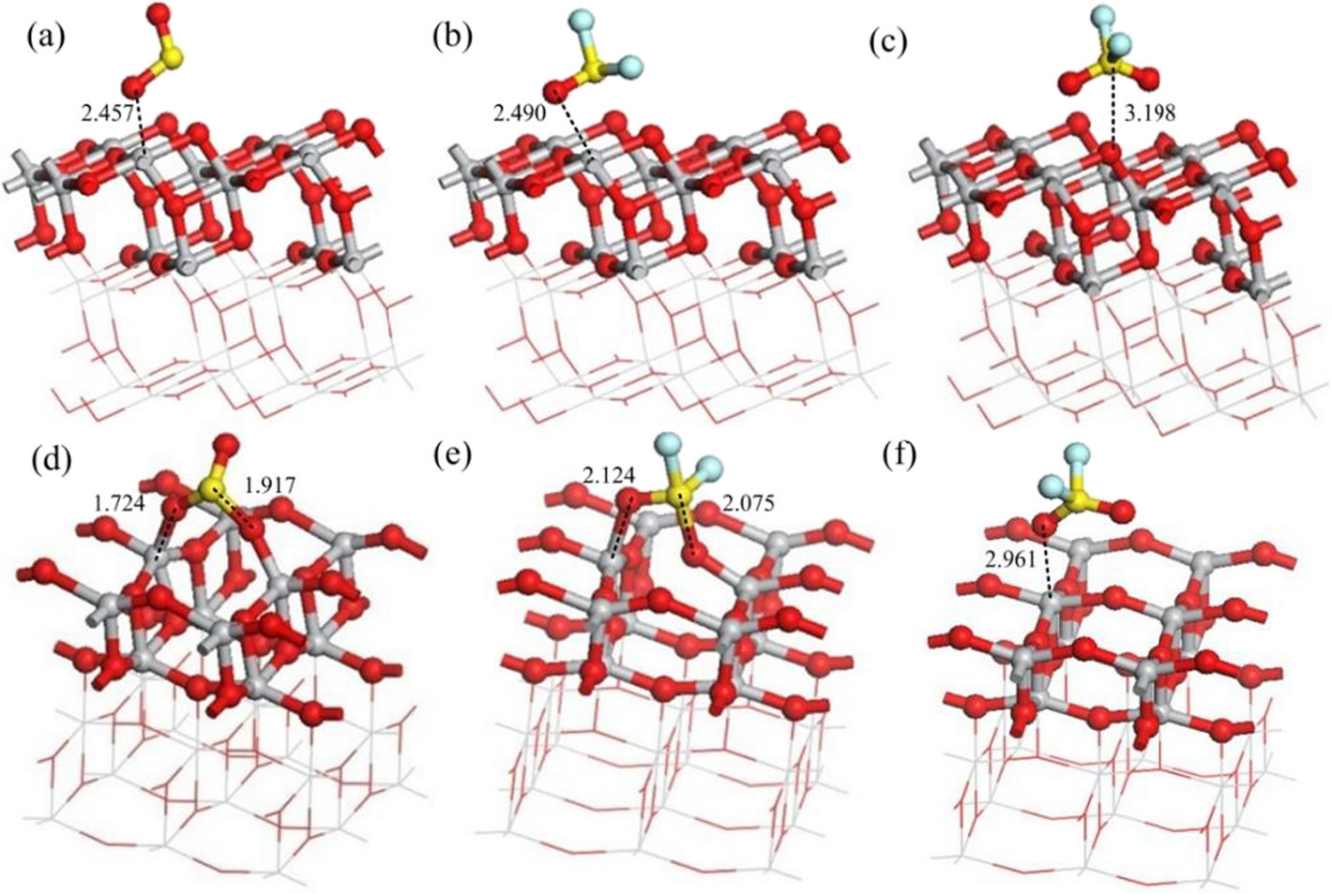
Adsorption structures of gas molecules on perfect surfaces: **a** SO_2_ adsorbs on the (1 0 1) perfect surface, **b** SOF_2_ adsorbs on the (1 0 1) perfect surface, **c** SO_2_F_2_ adsorbs on (1 0 1) perfect surface, **d** SO_2_ adsorbs on the (0 0 1) perfect surface, **e** SOF_2_ adsorbs on the (0 0 1) perfect surface, and **f** SO_2_F_2_ adsorbs on the (0 0 1) perfect surface. Binding distances are in Å

This section mainly discusses the theoretical studies employed to analyze the sensing mechanism to SO_2_, SOF_2_, and SO_2_F_2_. Xiaoxing’s group has conducted a lot of researches in online monitoring for SF_6_-insulated equipment by theoretical simulation method based on DMol^3^ module of materials studio [20, 22, 23, 41, 42]. It provides an effective way to explain the sensing mechanism at the molecular level by analyzing the adsorption energy, states of density, and energy band structure. Before gas molecules adsorption on the surface of TiO_2_, the structure of SO_2_, SOF_2_, SO_2_F_2_, and pure TiO_2_ are respectively optimized as shown in Figs. 2 and 3 [42]. As can be seen in Fig. 3, four surfaces, (1 0 1) perfect surface, (1 0 1) defect surface, (0 0 1) perfect surface, and (0 0 1) defect surface, are presented to analyze the different adsorption in detail. The PBE function is taken as the generalized gradient approximation to deal with the exchange-correlation energy in the whole simulation. To ensure the computation accuracy, the density functional computation adopts the double numerical basis set including p-polarization function. And the energy convergence, electronic self-consistent field, maximum force and displacement are respectively set as 1 × 10^−5^ Ha, 1 × 10^−6^ Ha, 2 × 10^−3^ Ha/Å, and 5 × 10^−3^ Å. The brillouin zone is sampled by 3 × 1 × 2 and 3 × 3 × 1 for (1 0 1) and (0 0 1) surface models, respectively [42]. According to the computation results of energy band, the computational value of energy gap (2.161 eV) is consistent with other computational results, though it is smaller than its experimental value (3.23 eV) (Fig. 4).

#### (1 0 1) and (0 0 1) Perfect Surface of TiO_2_

**Fig. 5:**
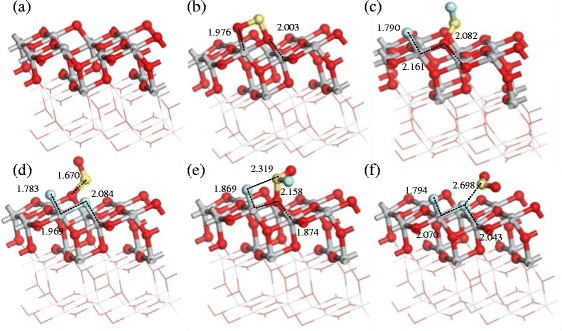
Adsorption structures of the gas molecules on the (1 0 1) defect surface: **a** The clear (1 0 1) defect surface, **b** SO_2_ adsorption, **c**, **d** SOF_2_ adsorption with different initial positions, and **e**, **f** SO_2_F_2_ adsorption with different initial positions. Binding distances are in Å

Figure 5 shows the adsorption structures of SO_2_, SOF_2_, and SO_2_F_2_ on the (1 0 1) and (0 0 1) perfect surface of TiO_2_ nanotubes [42]. The adsorption energy, charge transfer, binding distance, and energy gap are shown in Table 1. The adsorption energy and charge transfer decrease in the following order: SO_2_ > SOF_2_ > SO_2_F_2_ on both of the surfaces, respectively. But both of the adsorption energy and charge transfer on (0 0 1) surface are distinctly larger than that on (1 0 1) surface for all gas molecules, indicating that gas molecules are physisorbed on (1 0 1) surface and chemisorbed on (0 0 1) surface. Comparing the sensing properties to these three gas molecules, the (1 0 1) and (0 0 1) perfect surface of TiO_2_ show better adsorption property to SO_2_ than SOF_2_ and SO_2_F_2_.

**Table 1:** Calculated adsorption energy, charge transfer, and binding distance of the perfect surfaces

Surface	(1 0 1) perfect surface				(0 0 1) perfect surface			
Structure	TiO_2_ (1 0 1)	SO_2_	SOF_2_	SO_2_F_2_	TiO_2_ (0 0 1)	SO_2_	SOF_2_	SO_2_F_2_
*E*_*a*_ (eV)	**\**	−0.360	−0.297	−0.214	**\**	−1.660	−1.170	−0.690
Q (e)	**\**	0.097	0.051	0.010	**\**	−0.356	−0.118	0.014
d (Å)	**\**	2.457	2.490	3.198	**\**	1.724	2.075	2.961
*E*_*g*_ (eV)	1.951	1.788	1.932	1.936	1.613	1.488	1.565	1.602

#### Oxygen-Defect (1 0 1) Surface of TiO_2_

As shown in Fig. 3, different adsorption sites are discussed for SO_2_, SOF_2_, and SO_2_F_2_ adsorption on oxygen vacancy induced (1 0 1) defect surface of TiO_2_ [42]. The energy gap of (1 0 1) defect surface (1.888 eV) is distinctly smaller than that of (1 0 1) perfect surface (1.951 eV). For SO_2_ adsorption, one sulfur atom takes the place of oxygen vacancy and the other sulfur atom interacts with Ti atoms. When SOF_2_ adsorbs on (1 0 1) defect surface of TiO_2_, the F-S bond of SOF_2_ tends to break because of the strong chemisorption. Similarly, the F–S bond of SO_2_F_2_ breaks in the adsorption process seen in Fig. 3e, f. As a result, the conductivity of (1 0 1) defect surface increases after SO_2_ and SOF_2_ adsorption. Conversely, adsorption of SO_2_F_2_ increases the band gaps and reduces the conductivity of the (1 0 1) defect surface (Table 2).

**Table 2:** Calculated adsorption energy, charge transfer, and binding distance of (1 0 1) defect surface

Surface	(1 0 1) defect surface					
Structure	TiO_2_ (1 0 1)	SO_2_ (b)	SOF_2_ (c)	SOF_2_ (d)	SO_2_F_2_ (e)	SO_2_F_2_ (f)
*E*_*a*_ (eV)	\	−2.150	−2.095	−3.037	−4.356	−4.686
Q (e)	\	−0.699	−0.183	−0.734	−0.978	−0.877
d (Å)	\	1.976	1.790	1.670	1.869	1.794
*E*_*g*_ (eV)	1.888	1.524	1.250	1.283	1.932	1.935

#### Oxygen-Defect (0 0 1) Surface of TiO_2_

**Fig. 6:**
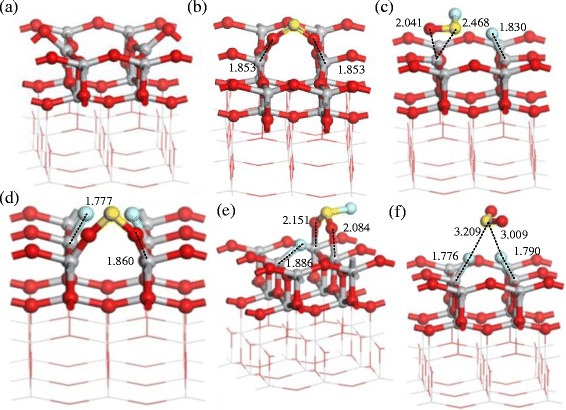
Adsorption structures of the gas molecules on the (0 0 1) defect surface: **a** The clear (0 0 1) defect surface, **b** SO_2_ adsorption, **c**, **d** SOF_2_ adsorption with different initial positions, and **e**, **f** SO_2_F_2_ adsorption with different initial positions. Binding distances are in Å

Figure 6 presents the adsorption structures of SO_2_, SOF_2_, and SO_2_F_2_ on the oxygen-defect (0 0 1) surface of TiO_2_ [42]. The sulfur atoms of SO_2_ molecule interact with the Ti atoms with bonding distance of 1.853 Å by physisorption as shown in Fig. 6b. When SOF_2_ and SO_2_F_2_ molecule adsorb on the surface with different initial positions as shown in Fig. 6c, d, the structures break because of the strong chemisorption. As the adsorption energy and charge shown in Table 3, the (0 0 1) defect surface of TiO_2_ shows stronger adsorption than (1 0 1) defect surface. The SO_2_ and SOF_2_ adsorption increase the conductivity of (0 0 1) defect surface by introducing the impurity state between valence and conduction band. While SOF_2_ adsorption leads to the decrease of conductivity of the (0 0 1) defect surface according to the widened energy gap.

**Table 3:** Calculated adsorption energy, charge transfer, and binding distance of the (0 0 1) defect surface

Surface	(0 0 1) defect surface					
Structure	TiO_2_ (0 0 1)	SO_2_ (b)	SOF_2_ (c)	SOF_2_ (d)	SO_2_F_2_ (e)	SO_2_F_2_ (f)
*E*_*a*_ (eV)	\	−3.205	−3.095	−4.810	−4.807	−4.786
Q (e)	\	−0.603	−0.701	−0.996	−0.978	−0.929
d (Å)	\	1.925	1.830	1.777	1.886	1.776
*E*_g_ (eV)	1.385	1.263	1.469	1.204	1.562	1.499

#### Pt-Decorated (1 0 1) Surface of TiO_2_

**Fig. 7:**
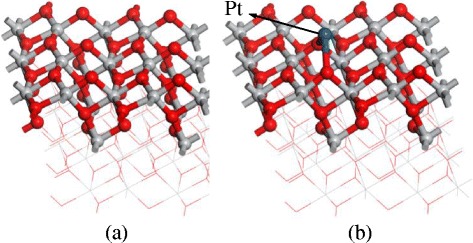
**a** (1 0 1) perfect surface of TiO_2_; **b** Pt-decorated (1 0 1) surface of TiO_2_

**Fig. 8:**
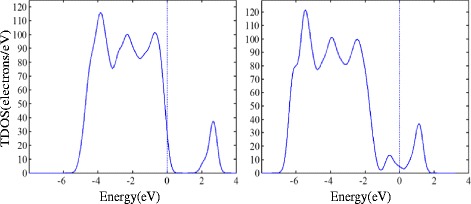
**a** DOS of TiO_2_ (1 0 1) perfect surface; **b** DOS of TiO_2_ Pt-decorated (1 0 1) surface

Pt atoms decoration TiO_2_ is widely used to enhance the gas response in different gas detection field. In this section, we present the theoretical computation of Pt-decorated (1 0 1) surface of TiO_2_ and its gas response to SO_2_, SOF_2_, and SO_2_F_2_. As the structure of pure and Pt-decorated (1 0 1) surfaces of TiO_2_ shown in Fig. 7, the Pt atom builds a stable structure with two oxygen atoms. Figure 8 shows the density of states (DOS) of (1 0 1) perfect surface and Pt-decorated (1 0 1) surface of TiO_2_. It is found that the separated valence and conductive bond become continuous after Pt decoration, signifying the increasing conductivity of TiO_2_ (1 0 1) surfaces.

**Fig. 9:**
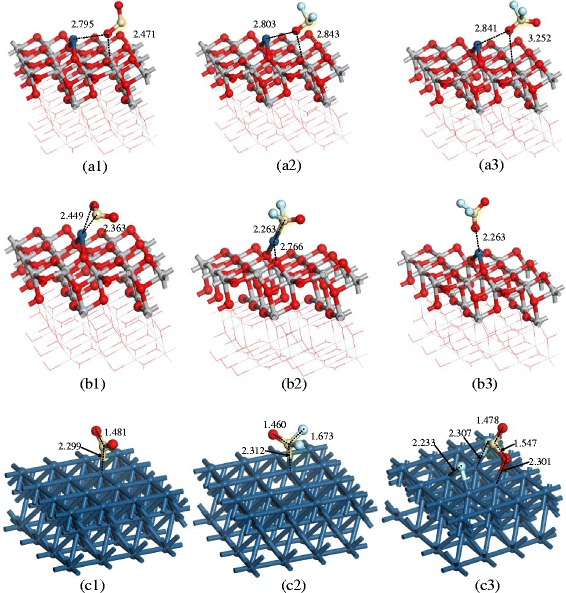
Adsorption structures of the gas molecules on different sites of Pt-decorated (1 0 1) surface (**a1**)-(**a3**) adsorption of SO_2_, SOF_2_ and SO_2_F_2_ on Pt doped TiO_2_ (1 0 1) perfect surface away from Pt atom, (**b1**)-(**b3**) adsorption of SO_2_, SOF_2_ and SO_2_F_2_ at the boundary between Pt atom and TiO_2_ (1 0 1) perfect surface, (**c1**)-(**c3**) adsorption of SO_2_, SOF_2_ and SO_2_F_2_ on Pt atoms

Considering that Pt decoration on the surface of TiO_2_ (1 0 1) is usually in the form of Pt particles, three different adsorption situations for SO_2_, SOF_2_, and SO_2_F_2_ are discussed in Fig. 9. Figure 9a1–a3 show the adsorption of SO_2_, SOF_2_, and SO_2_F_2_ on the surface of TiO_2_ (1 0 1) perfect surface away from Pt atom. The Pt decoration brings little influence to the adsorption of SO_2_, SOF_2_, and SO_2_F_2_ molecules; three molecules are physisorbed on the TiO_2_ (1 0 1) perfect surface, indicating that the enhancement of gas sensing comes from the adsorption around Pt atoms. Figure 9b1–b3 present the adsorption of SO_2_, SOF_2_, and SO_2_F_2_ at the boundary between Pt atom and TiO_2_ (1 0 1) perfect surface. The Pt aom acts as the active site to adsorb the SO_2_, SOF_2_, and SO_2_F_2_ molecules. SO_2_ and SOF_2_ prefer to approach the Pt atom by sulfur atom with nearest binding distances of 2.363 and 2.263 Å, respectively, while SO_2_F_2_ approaches to the Pt atom by oxygen atom of SO_2_F_2_. For SO_2_, SOF_2_, and SO_2_F_2_ adsorption on the surface of Pt particles, a (2 0 0) surface of Pt metal is considered in the study as shown in Fig. 9c1–c3; the SO_2_ and SOF_2_ molecules interact with Pt atom with distances of 2.299 and 2.312 Å. And the interaction between (2 0 0) surface of Pt metal and SO_2_F_2_ promotes the decomposition of SO_2_F_2_.

### Experimental Observations

#### Pure TiO_2_

In this section, the gas response (change of resistance) of TiO_2_ nanotube arrays (TNTAs) to SO_2_, SOF_2_ and SO_2_F_2_ are discussed at 200 °C, and the negative value of resistance variation (R%) means the reduction of resistance. According to the correspondence between gas response and concentration, it is found that the change between them presents a fitting curve relationship. As a result, we can directly estimate the concentration of gas according to the corresponding gas response.

**Fig. 10:**
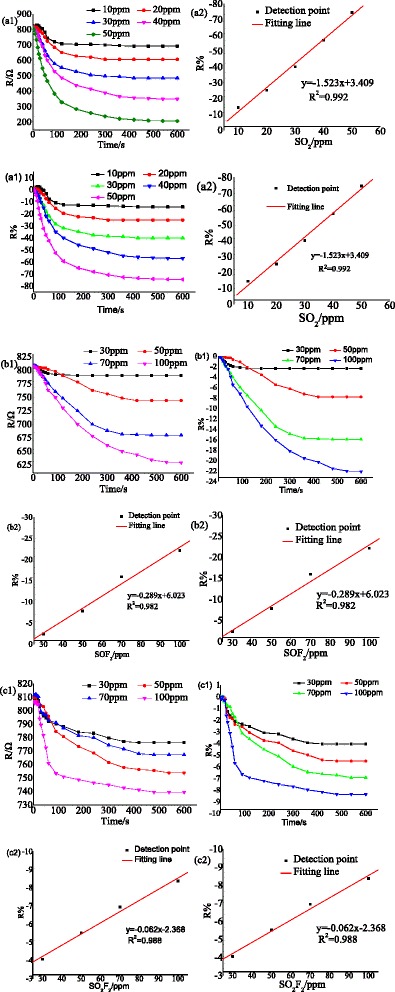
Pure TNTAs response to different SF_6_ decomposition products at 200 °C. **a1**, **a2** SO2 sensing, **b1**, **b2** SOF_2_ sensing, and **c1**, **c2** SO_2_F_2_ sensing

Figure 10a1, a2 show the gas response to SO_2_ with different concentration: 10, 20, 30, 40, and 50 ppm [40]; the horizontal and vertical ordinate present the gas sensing time and gas response, respectively. It is found that the resistance of TNTAs rapidly decreases when it contacts with SO_2_ and eventually reaches a stable value with time. The SO_2_ response to 10, 20, 30, 40, and 50 ppm are −14.35, −25.23, −40.16, −57, and −74.6 %, respectively. And a fitting line: *y* = − 1.523*x* + 3.409 with fit goodness of 0.992 is obtained. According to the change of resistance shown in Fig. 10b1, b2, the resistance decrease after SOF_2_ detected by TNTAs at 200 °C. In addition, the gas sensing time increase with the concentration with SO_2_. The response to 30, 50, 70, and 100 ppm SOF_2_ are −2.38, −7.82, −15.95, and −22.13 %, respectively. And the fitting line is *y* = − 0.289*x* + 6.023 with fit goodness of 0.982. Although the resistance of TiO_2_ nanotubes array decreases during SO_2_F_2_ contacting, the change of resistance is obviously smaller than that of SO_2_ and SOF_2_ sensing at the same gas concentration and temperature. The highest gas response is only −8.37 % when the concentration of SO_2_F_2_ reaches 100 ppm. The fitting line is *y* = − 0.062*x* − 2.368 with fit goodness of 0.988.

Comparing the gas sensing speed and magnitude of resistance change at the same gas concentration and temperature, the gas response of TNTAs to SF_6_ decomposition products is in orders, SO_2_ > SOF_2_ > SO_2_F_2_, indicating the potential of selective detection between SO_2_, SOF_2_, and SO_2_F_2_.

#### Pt-Decorated TNTAs

**Fig. 11:**
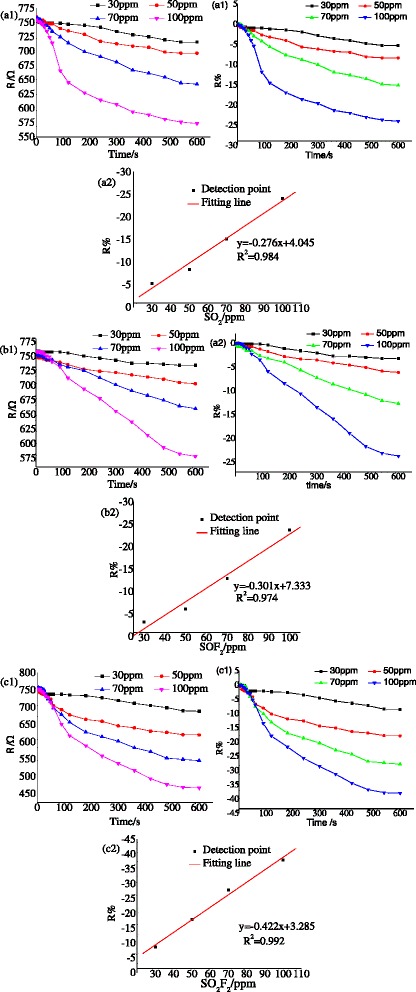
Pt-TNTAs response to different SF_6_ decomposition products at 150 °C. **a1**, **a2** SO_2_ sensing, **b1**, **b2** SOF_2_ sensing, and **c1**, **c2** SO_2_F_2_ sensing

Figure 11 presents the gas sensing property of Pt-decorated TNTAs (Pt-TNTAs) to SO_2_, SOF_2_, and SO_2_F_2_ at 150 °C [40, 43]. Due to the decoration of Pt particles on the surface of TiO_2_ nanotubes, it not only enhances the gas response to SOF_2_ and SO_2_F_2_ also reduces the working temperature for gas detection. As shown in Fig. 11a1–a2, the gas response to different concentrations of SO_2_, 30, 50, 70, and 100 ppm are −5.31, −8.38, −15.18, and −24.07 %. After linear fitting, the corresponding relation between SO_2_ concentration and gas response is *y* = − 0.276*x* + 4.405 with fit goodness of 0.984. The change of resistance of Pt-TNTAs is obviously smaller than that of pure TNTAs in the SO_2_ detection process. For instance, the gas response of pure TNTAs and Pt-TNTAS is −74.6 and −8.38 % when the concentration of SO_2_ is 50 ppm. As shown in Fig. 11b1–b2, the response of Pt-TNTAs to 30, 50, 70, and 100 ppm SOF_2_ are 3.23, −6.11, −12.92, and −23.75 %, respectively. And the fitting line is *y* = − 0.301*x* + 7.333, indicating that the change of response to different concentration of SOF_2_ is still linear. The response to SOF_2_ slightly increases comparing with that of pure TNTAs at the same SOF_2_ concentration. Therefore, the improvement for SOF_2_ detection from Pt decoration mainly reflects on the aspect of the working temperature reduction. As the gas response of Pt-TNTAs to SO_2_F_2_ shown in Fig. 11c1–c2, the change of resistance for 30, 50, 70, and 100 ppm of SO_2_F_2_ are −8.65, −17.91, −27.86, and −38.02 %. And a liner function (*y* = − 0.422*x* + 3.285) is received with high fit goodness of 0.992. Comparing the gas response before and after Pt decoration, it is found that the gas response distinctly increases at the same SO_2_F_2_ concentration, indicating that the Pt-TNTAs possesses selective detection for SO_2_F_2_ after Pt particles decoration.

#### Au-Decorated TNTAs

**Fig. 12:**
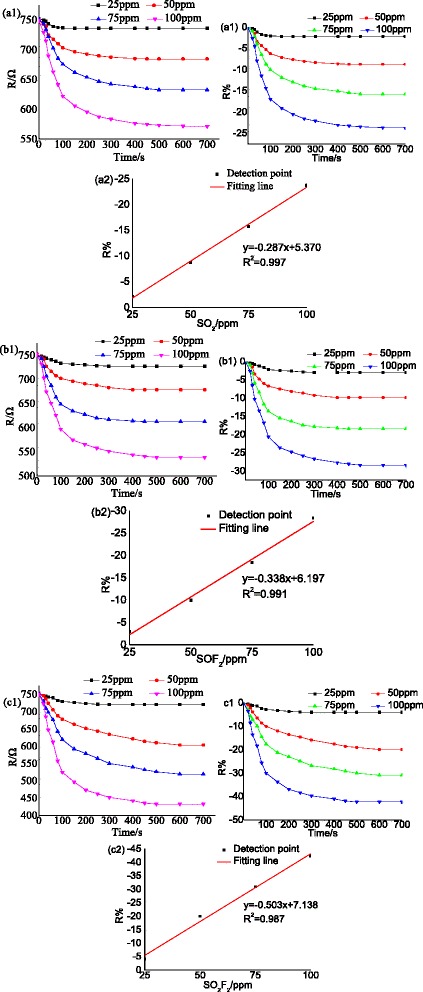
Au-TNTAs response to different SF_6_ decomposition products at 110 °C. **a1**, **a2** SO_2_ sensing, **b1**, **b2** SOF_2_ sensing, and **c1**, **c2** SO_2_F_2_ sensing

The gas response of Au-decorated TNTAs (Au-TNTAs) to SO_2_, SOF_2_ and SO_2_F_2_ are discussed in this section as shown in Fig. 12. The working temperature (120 °C) applied in the detection process is much lower than that of pure TNTAs and Pt-TNTAs sensors, which is benefit for wide spread application. Fitting the resistance with different concentration of SO_2_, SOF_2_, and SO_2_F_2_ (25, 50, 75, and 100 ppm) shown in Fig. 12a2–c2, the response of Au-TNTAs to different kinds of SF_6_ decomposition components is in the following order: SO_2_F_2_ > SOF_2_ > SO_2_. Comparing the gas response property of pure TNTAs, Pt-TNTAs and Au-TNTAs sensors to SF_6_ decomposition components, metal decoration not only enhances the gas response to the decomposition components but also realizes the selective detection to different decomposition components. In addition, metal decoration effectively reduces the working temperature.

## Conclusions

TiO_2_ nanotube arrays (TNTAs) has been widely used as gas sensor for its distinguished properties in large specific surface area, large pore structure, easy synthesis process, and environmentally friendly nature. In order to evaluate and diagnose the insulation status of SF_6_-insulated equipment, TNTAs gas sensor becomes an effective new method to realize the function by detecting the decomposition components of SF_6_: SO_2_, SOF_2_, and SO_2_F_2_. In terms of TNTAs synthesis, three methods, assisted-template method, hydrothermal treatment method, and anodization method, are discussed to analyze the preparation process and the features of prepared TNTAs in detail. Then, recent studies carried out by theoretical simulation have been viewed. The adsorption of SO_2_, SOF_2,_ and SO_2_F_2_ on different surface of TiO_2_ is reviewed in this section, including (1 0 1) and (0 0 1) perfect surface of TiO_2_, oxygen-defect (1 0 1) and (0 0 1) surface of TiO_2_, and Pt-decorated (1 0 1) surface of TiO_2_. Finally, the experimental researches used to analyze the gas response of TNTAs sensor to SO_2_ and SOF_2_ and SO_2_F_2_ are discussed. Comparing the gas response to SO_2_, SOF_2_, and SO_2_F_2_ by different gas sensors (pure TNTAs sensor and Pt, Au-decorated TNTAs sensor), it is found that the metal decoration improves the gas response property to SO_2_ and SOF_2_ and SO_2_F_2_ and also reduces the working temperature for gas detection. Further, more studies should be investigated to enhance the detection precision and stability of TNTAs, aiming to industrialize the fabrication and application of TNTAs sensor in SF_6_-insulated equipment.
